# Round the Hospitals

**Published:** 1921-01-08

**Authors:** 


### ROUND THE HOSPITALS.

The New Year Honours' List contains several names which cannot fail to interest nurses. It is dealt with, so far as hospital and cognate interests are concerned, on page 329, and in addition we have to chronicle the award of a Bar to the Royal Red Cross to Miss Dorothea Matilda Taylor, R.R.C., Acting Principal Matron, Q.A.I.M.N.S., and of the Royal Red Cross, Second Class, to Miss Lizzie Parrish Dixon, Matron, Military Families Hospital Staff.

---

The announcement in our advertisement columns last week relating to the College scholarships at Halifax is worthy of attention It offers a free course of midwifery to be competed for by entrants who are College members, and who must either have been trained at the Royal Halifax Infirmary or at St. Luke's Hospital, or been actively nursing in the town of Halifax for at least one year. The scholarship is offered by the Halifax Branch of the Yorkshire Centre, and suggests a valuable development of the scholarships movement now making itself felt in the nursing world. Time was when the nurse in training and after training had little stimulus to advance her in her profession. It begins to be clear that the College is exercising a great influence in raising the standard of education, and that the centres and their branches are strongly reacting in this direction. Such spontaneous acts of generosity on the part of individual branches is good, not only for the nurses who compete, but also for the College at large. It is a healthy sign of corporate responsibility and good fellowship.

It is generally recognised by those who have to deal with the problem of the supply of trained nurses that the surest way to attract the right women to the nursing profession is to raise the standard of education of its members. This point of view is being given practical expression at the best training-schools throughout the country, where opportunities are afforded to graduates to qualify in additional branches of their profession. At Chelsea Infirmary Miss Barton has inaugurated a new scheme, by which six of the best nurses at the end of their third year may take their midwifery training and qualify for the certificate of the Central Midwives Board. Two of the Chelsea nurses have just started at the East End Mothers' Home, and when they return to their training-school to finish their fourth year two others will take their place. It is hoped to extend this scheme later, and it is hardly necessary to say that its introduction is much appreciated by the Chelsea Infirmary graduates.

The spring term of the Royal Sanitary Institute commences the first week in February, and embraces separate courses of lectures for the training of sanitary officers, meat and food inspectors, health visitors, school nurses, and maternity and child-welfare workers. Full particulars appear in our advertising columns. In addition to the book work in preparation for the examinations of the Institute, many valuable practical demonstrations and visits to public works have been arranged for the students, who also have the use of a fine reference library and reading-room. The inspections include such useful and interesting visits as demonstrations of bookkeeping as carried out in a sanitary inspector's office; on meteorological instruments in the Parkes Museum; of L.C.C. municipal lodging-house; of Express Dairy Company's farm at Finchley, with visits to margarine factories, soapworks, etc. We wish it might be possible for hospitals to allow duly qualified entrants to attend the course for health visitors during their third or fourth year, so that on the expiration of their term of hospital training they could pass on at once to public health work. This is done in America, and we believe it is bound to come in this country. The introductory lecture will be given by Professor H. R. Kenwood at the Institute on January 31, at 5.30. Admission free.

The National Association for the Prevention of Infant Mortality and for the Welfare of Infancy, with its companion, the National Society of Day Nurseries, has arranged good courses of lectures in the ensuing months. The elementary course of lectures on infant care for crèche nurses and probationers commences on January 20. A more advanced course for infant-welfare workers, teachers, and mothers commences on January 17. Full particulars can be obtained from Miss Halford, Secretary, 4/5 Tavistock Square, W.C. 1. We could wish the combined societies would hit upon a less unwieldy name. As it stands it lends a rather absurd air of the ponderous to the lightest allusion to their activities. We are interested to see that lectures on infant care are also to be given at the Victoria Institute, Worcester.

Miss S. Riddell, Registrar for the General Nursing Council for England and Wales, has addressed a circular letter bearing her signature, addressed from the Ministry of Health, Whitehall, to boards of guardians enclosing an elaborate schedule asking that the information required should be supplied. These particulars, she stated, were sought by the Council in order that they may be assisted in drawing up the rules for the education and examination of nurses in the future. When this letter was read at the meeting of the Southwark Board of Guardians, the Clerk (Mr. Stanwell Smith) pointed out that requests were sent out personally in the name of Dr. Addison, and were signed by him. The guardians decided that as the information required would take a considerable time to collect the Clerk should write to Dr. Addison asking if the letter was sent out at his instigation and whether the information required should be supplied. The Southwark Board is merely wasting time and making work by such a reply. Quite obviously it is the duty of the General Nursing Council to collect information from all the training-schools before making rules which must so closely affect their interests, and the Registrar of the Council is an official all will have to learn to respect.

Something like consternation descended upon the Devonport Board of Guardians at their meeting shortly before Christmas owing to a rumour that, after the lapse of two years, no nurse will be allowed to sit for the central examination unless she has been trained in an institution containing at least 200 beds. This announcement was made by the Clerk (Mr. Albert Gard), who stated that he and the Chairman of the Infirmary Committee had lately had an interview with Dr. Fuller, of the Ministry of Health, and had received the impression that after November 1922 entrants for examination would have to be qualified by four years' training in institutions, of which three would have to be spent in an institution containing not less than 200 beds under a resident medical officer. The Clerk stated that there was hardly a hospital in the West of England where nurses could be trained were these conditions to be imposed. They were informed that they might still have a superintendent nurse and engage women and girls to help in the nursing, but such assistants would never become nurses. These alarming statements were subsequently denied authoritatively at the Ministry of Health and stigmatised as an absolutely wild proposition which might do serious harm unless promptly denied. Miss Kearsey, Hon. Secretary of the Plymouth Branch of the College of Nursing, has pointed out to the Press the incoherency of the suggestions retailed by the Clerk, and explained that it is hoped the case of small hospitals will be met by an affiliation scheme now under consideration. The sooner the General Nursing Council issues its rules the better for all concerned.

A NEW and very flourishing institution is the Chelsea Nurses' Club for the nurses of the Chelsea Hospital for Women, the Brompton Hospital, Chelsea Infirmary, Cancer Hospital, and Victoria Hospital for Children. It has been formed to give the members facilities for recreation and amusement. Meetings take place at the different hospitals in turn, and this interchange of visits does much to contribute to the vigour of the organisation. It is hoped that in the summer tennis and badminton may be added to the games list. The members of the Club have recently distinguished themselves by a clever dramatic and musical entertainment given at the Chelsea Hospital for Women.

THE excessive hours on duty at the Portsmouth Infirmary have given rise to much discontent, and what is popularly termed unrest, in the institution. The Infirmary Committee has recommended the engagement of ten extra probationers, by means of which it is proposed to reduce the hours on duty to fifty-six in the week. Eleven cubicles for the accommodation of the nursing staff are to be erected at a cost £470. The Guardians have assented to both these recommendations. But since the urgently-needed reform forecasted can hardly be carried out until the cubicles are in being, and since the engagement of ten additional probationers may prove less easy in the present state of labour than it sounds, there should be some preliminary relief granted so as to tide over the period of unrest. Would it not be reasonable to grant bonuses in the form of overtime grants to nurses kept on duty more than fifty-six hours a week?

THE *London Gazette* of December 31 gives notice that in pursuance of the Regimental Debts Act, 1893, there is available for distribution amongst the next-of-kin or others entitled certain sums of money which are set out against the names of deceased officers, soldiers, and airmen, lists of which are given. Copies of these lists, which include the names of sisters and nurses of Q.A.I.M.N.S., the Territorial Force, V.A.D., the Women's Legion and other services are to be seen at the Regimental Depôts and Head Post Offices throughout the United Kingdom. Applications from persons supposing themselves entitled as next-of-kin should be addressed by letter to the Secretary, War Office, London, S.W. 1, and marked outside "Effects."

THE Jabberwock's Party at the National Hospital, Queen's Square, on New Year's Day was a notable affair. Every member of this delightful Association, which was started ten years ago by Miss Brenda Girvin, is pledged to send 6d. and a toy every Christmas to children's hospitals under their ægis. They began first with the National Hospital, but have now spread far and wide, and number nearly 1,200. Princess Christian, accompanied by Princess Marie Louise, gave unwonted festivity by her presence on this occasion. The Royal party was received by the Chairman, Miss Spackman, the matron, and members of the medical staff, and tea was served in the board-room on their arrival to a large company. The patients' hall was brilliantly decorated for the occasion, and a magnificent Christmas-tree, the annual gift of a Covent Garden dealer whose daughter was once a patient, held the place of honour. Princess Christian helped to distribute the gifts, and a capital entertainment wound up the proceedings.

SUNDAY, April 24, 1921, will be an important occasion for all of His Majesty's subjects. At midnight on that day a Census is to be taken for Great Britain, and directions have been published with respect to the persons by whom and to whom the returns are to be made, and the particulars to be included. In the case of persons present at midnight on the Census Day on the premises of any public or private hospital, sanatorium, convalescent or nursing home, workhouse, poorhouse, infirmary, asylum, religious or charitable community, residential school or college, or residential institution of any kind, the chief resident officer, or other person for the time being in charge, is required to make the return. This will frequently be the matron or lady superintendent. Amongst the particulars to be supplied in the return is whether an individual is entitled to medical benefit under the National Insurance (Health) Acts.

A DISTRESSING occurrence took place at the Whipps Cross Hospital on December 16, when one of the maids, Cissie Gasson, aged twenty, set fire to her nightdress and received burns which resulted in her death. She returned early in the evening from a day's leave, and in making herself some coffee at an open fire after getting ready for bed she was seen to be in flames. Edith Vaughan, one of her room mates, pluckily tried to wrap a mat round her but she tore herself away and ran down the ward. Miss Vaughan pursued and again clung desperately to her, wrapping round her a wet garment, and at length extinguished the flames quite regardless of her own danger. Unhappily by this time severe burns had been inflicted on the face and arms and trunk which resulted in death twenty-four hours later. At the inquest held before Dr. Badenoch, the Coroner highly commended Miss Vaughan for her courage and recommended her for notice to the Society for the Protection of Life from Fire. It would seem that she has imbibed no small share of the nursing spirit in presence of danger during her residence in the infirmary.

